# Evaluation of Techniques for Intensifying the Process of the Alcoholic Extraction of Coffee Ground Oil Using Ultrasound and a Pressurized Solvent

**DOI:** 10.3390/foods11040584

**Published:** 2022-02-17

**Authors:** Tatiane Akemi Toda, Ana Julia Morelli Santana, Julieta Adriana Ferreira, Eliria Maria de Jesus Agnolon Pallone, Claudio Lima de Aguiar, Christianne Elisabete da Costa Rodrigues

**Affiliations:** 1 Laboratório de Engenharia de Separações (LES), Departamento de Engenharia de Alimentos (ZEA), Universidade de São Paulo (USP), P.O. Box 23, Pirassununga 13635-900, Brazil; tatiane.toda@usp.br (T.A.T.); ana.julia.morelli@usp.br (A.J.M.S.); 2 Departamento de Engenharia de Biossistemas (ZEB), Faculdade de Zootecnia e Engenharia de Alimentos (FZEA), Universidade de São Paulo (USP), P.O. Box 23, Pirassununga 13635-900, Brazil; julieta.ferreira@usp.br (J.A.F.); eliria@usp.br (E.M.d.J.A.P.); 3 Departamento de Agroindústria, Alimentos e Nutrição, Escola Superior de Agricultura “Luiz de Queiroz” (ESALQ), Universidade de São Paulo (USP), Piracicaba 13418-900, Brazil; claguiar@usp.br

**Keywords:** UAE, PLE, PCA, coffee byproduct, instant-coffee residue, green solvent, multistage cross-current extraction

## Abstract

Ultrasound-assisted extraction (UAE) and pressurized liquid extraction (PLE) techniques were evaluated and compared with conventional extraction for obtaining spent coffee ground oil (SCGO). The use of absolute ethanol (ET0) and hydrated ethanol (ET6) as solvents, two levels of SCG mass ratio:solvent, 1:4 (U4) and 1:15 (U15), and ultrasound powers of 0, 200, 400, and 600 W were tested. ET0 and U15 resulted in higher extraction yields of SCGO (Y_SCGO_, 82%). A positive effect of sonication on Y_SCGO_ was observed only for condition U4. UAE resulted in defatted solids (DS) with higher apparent density values, corroborating the increase in the amount of smaller diameter particles due to sonication. The micrographs showed changes in the surfaces of the solids from the UAE and PLE, although the crystalline structures of the DS were not altered. UAE and PLE, compared to conventional extraction, did not allow significant gains in terms of Y_SCGO_ and, consequently, in the number of contact stages in an extractor configured in cross-currents.

## 1. Introduction

Coffee is one of the most popular and consumed beverages in the world, with significant cultural, economic, and social importance [1]. According to the United States Department of Agriculture [2], between 2020 and 2021, 175.8 million 60 kg bags of coffee beans were produced worldwide. The main residue from the consumption of this beverage is spent coffee grounds (SCG), which have a considerable content of lipids (7 to 21%) and high value minor compounds that can be obtained from spent coffee ground oil (SCGO) by fractionation and purification processes [3].

The worldwide consumption of vegetable oils has increased every year [2] in parallel with a growing search for new sources of renewable energy motivated by the need to mitigate global warming [4]. These circumstances reinforce the importance of the search for other unconventional sources of vegetable oils, such as SCG. In this context, several research groups have proposed ways to add value to SCG through the extraction of SCGO [5,6,7].

To mitigate pressure on the global ecosystem, the minimization of the use of compounds derived from fossil sources has received attention. In the context of vegetable oil extraction, the challenge is to replace hexane, a solvent traditionally used in industrial practice. The suggested substitute is ethanol, a less polluting solvent, with low toxicity and greater operational safety [8,9,10]. In an economic, technical, and environmental analysis performed by Potrich et al. [8], the authors found that the use of ethanol in the extraction of soybean oil is economically viable. However, it does not have the same profitability as hexane extraction.

The advancement and improvement in emerging extraction technologies such as microwave-assisted extraction, supercritical fluid extraction, pressurized liquid extraction (PLE), and ultrasound-assisted extraction (UAE) can intensify the solid–liquid extraction process, making it more advantageous than conventional systems.

PLE is considered a green and very efficient technology for the extraction of compounds in short periods of time. In this technique, the pressure favors the penetration of the solvent into the solid matrix [11]. Araújo et al. [12] obtained higher values of extraction yield of SCGO with pressurized ethanol than the yields obtained by Soxhlet extraction using hexane as a solvent. Muangrat and Pongsirikul [13] and Efthymiopoulos et al. [6] obtained higher extraction yields of SCGO with PLE than with Soxhlet extraction. One of the main advantages of PLE is the speed of the extraction reaching a steady state. In addition, PLE allows the use of less solvent [14]. Toda et al. [14], using ethanol as the solvent, found that the conventional extraction of SCGO would require 20 to 30 min to reach steady state. With the use of pressurized ethanol, the extraction time was reduced by half, requiring 2 to 15 min to obtain more concentrated SCGO extracts.

The application of ultrasound in the extraction processes is used to break the cell walls of the solid matrix and thus to favor the arrangement of the oil in the liquid, improving the diffusion of this compound between the solid and solvent phases [15,16]. Cubas et al. [17] obtained an increase in the extraction yield of SCGO using an ultrasound-assisted treatment before the Soxhlet extraction step. Okur et al. [18] observed a higher extraction yield of phenolic compounds from SCG when using probe ultrasound. Rocha et al. [19] reported the influence of the ratio between hexane and SCG and the application of ultrasound on the increase of the effective diffusivity of SCGO in the liquid phase. Goh et al. [4] also obtained greater SCGO extraction with the application of ultrasound than with Soxhlet extraction using hexane, methanol, and chloroform as solvents.

Sicaire et al. [20] obtained higher extraction yields of canola oil using ultrasound with 7.7 W·cm^−2^ transmitted power intensity than using conventional extraction with hexane as a solvent. For the conventional extraction, three contact stages were necessary to exhaust the raffinate phase in a cross-current extractor. With the application of ultrasound, only two contact stages would be necessary. According to the authors, the application of ultrasound can provide a significant improvement in the processes of the extraction of vegetable oils.

Based on this information, the main objective of this study was to evaluate different techniques for intensifying the process of extracting SCGO using ethanol as the solvent. Extractions with pressurized ethanol and ultrasound-assisted ethanol in batch and fixed bed columns considering two different SCG:ethanol ratios (1:4 and 1:15), levels of solvent hydration (absolute ethanol (ET0) and hydrated ethanol (ET6)), and ultrasound powers (0, 200, 400, and 600 W) were evaluated in terms of SCGO extraction yield. The solids resulting from the extraction process were compared to SCG regarding morphology in terms of true and apparent density, particle size distribution, and mean particle diameter, in addition to crystallinity and scanning electron microscopy information. In addition, the design of extractors configured in cross-currents was performed, and it was possible to compare the different intensification techniques regarding the number of stages required to extract the oil from the SCG.

## 2. Materials and Methods

### 2.1. Materials

The raw material used for the development of this work is one of the co-products of the production of soluble coffee and spent coffee grounds (SCG). This material was supplied by a Brazilian coffee processing company (Araras, SP, Brazil). Before carrying out the extraction experiments, the SCG was subjected to drying (forced convection oven, Nova Orgânica, 35/3, Piracicaba, SP, Brazil) at 45 °C, for 4 h 20 min, to prevent the material degradation due to its high initial moisture (62.02 ± 0.05%). For SCGO extraction, absolute ethanol (ET0, purity ≥ 99.9%, CAS 64-17-5) (Merck, Darmstadt, Germany) and hydrated ethanol (ET6, with 6.0 ± 0.5 mass% water) were used as solvents. ET6 was prepared by adding deionized water (Millipore, Mili-Q, Bedford, MA, USA) to ET0, monitoring the water content in solvents and extracts through Karl Fischer titration (Ca 2e-84, [21]) (Metrohm, 787 KF Titrino, Herisan, Switzerland).

### 2.2. Physical Chemical Characterization of SCG

The chemical composition of SCG was determined following the protocols described by AOCS [21] in terms of lipids (Am 5-04), using a high-temperature oil extraction system (Ankom, XT 10, Macedon, NY, USA); crude protein (Ba 4f-00) in a combustion nitrogen determination system (Leco, FP-528, St. Joseph, MI, USA), considering 6.25 as a nitrogen-protein conversion factor; and moisture (Ac 2-41). The contents of ash [22], soluble and insoluble fibers [23], and the contents of hemicellulose, cellulose, and lignin [24,25] were also determined. All determinations were carried out at least in duplicate.

The size distribution of the SCG particles was evaluated by laser diffraction (Shimadzu, SALD-201 V, Kyoto, Japan) and sieves (Tyler series, Wheeling, WV, USA). Initially, the SCG was sieved for five minutes with manual shaking and then each sample mass retained in each sieve and the bottom tray was weighed. Samples from the bottom tray, with particles from 0.25 to 290 µm, were taken for analysis in laser diffraction, using petroleum jelly to disperse the particles.

The mean particle diameter ($d_{s})$, calculated by the particle size distribution using the sieves, was determined according to the ASAE methodology [26] (Equation (1)).

(1) $$d_{s}=\;log^{-1}\left[\frac{\sum_{i=1}^{n}m_{i}log{\left(d_{i}\times d_{i+1}\right)}^{0.5}}{\sum_{i=1}^{n}m_{i}}\right]$$

where $m_{i}$ is the mass retained on the *i*th sieve (g) and $d_{i}$ is the pore size of the *i*th sieve (mm).

The mean diameter of the bottom particles (bottom tray of Tyler sieves) ($d_{ld})$ was determined by laser diffraction and provided by the equipment itself, using Equations (2) and (3).

(2) $$d_{ld}=10^{\mu }$$

(3) $$\mu =\frac{1}{100}\overset{n}{\underset{i=1}{\sum }}q_{i}\frac{log_{10}x_{i}+log_{10}x_{i+1}}{2}$$

where $x_{i}$ is the particle diameter (µm) and $q_{i}$ is the differential distribution (%).

After determining the mean particle diameters for each methodology, ($d_{s})$ e ($d_{ld})$, the weighted average, was calculated, obtaining the mean particle diameter of the SCG ($d_{avg})$.

The true density ($\rho_{t})$ was determined by the pycnometry method using helium gas (Quantachrome Instruments, MVP-6DC, Delray Beach, FL, USA), and the apparent density ($\rho_{a})$ was determined by weighing on an analytical balance (0.0001 g, Adam, model PW 254, Milton Keynes, UK) of SCG mass that occupied a preset volume of a flask, previously calibrated with water, with the help of a bench digital densitometer (Anton Paar, model DMA 4500, Graz, Styria, Austria).

SCG characterization by scanning electron microscopy (SEM) (FEI Company, Inspect S50, Hillsboro, OR, USA) with tungsten filament at 15 kV acceleration was performed with a magnification of ×3000 under a high vacuum. Material previously dried in an oven with forced convection (130 °C, 3 h) and coated with a thin layer of gold was used.

SCG was also evaluated by X-ray diffraction (Rigaku, MiniFlex 600, Tokyo, Japan) operating with Cu-K alpha radiation, a voltage of 40 kV, and a current of 15 mA. The samples were analyzed from 5° to 90°, with a speed of 2° per minute. Baselines of all XRD diffractograms were fitted. Analytical deconvolution curves of all diffractograms were obtained using the Gaussian function (R2 > 0.999), with the Savitzky–Golay function used as a filter. All mathematical treatment was carried out in the OriginLab^®^ software (version 2020 Academic, OriginLab Corporation, Northampton, MA, USA). The peaks were compared with data from the International Center for Diffraction Data (ICDD) in the Crystallographica Search Match software (Oxford Cryosystems, CSM, Oxford, UK). The relative crystallinity of SCG was estimated by the percentage crystalline area concerning the total area (percent crystalline area + percentage amorphous area).

### 2.3. SCGO UAE

SCGO UAEs were performed with the aid of a VCX-750 HV ultrasound probe (Sonics and Materials, Newtown, CT, USA) with a titanium probe with a diameter of 25 mm, maximum power of the equipment of 750 W, and constant frequency of 20 kHz. These experiments were performed considering two configurations: batch, using a Pyrex glass cell jacketed and connected to a thermostatic bath; and in a fixed bed column system with extract recirculation. In the column configuration, a continuous flow cell made of stainless steel, jacketed and connected to a thermostatic bath (high volume continuous flow cell, Sonics and Materials, Newtown, CT, USA), was used. For both configurations, ET0 and ET6 were used as extraction solvents. The extraction experiments were performed at least in duplicate.

Considering that the energy of the ultrasound waves is converted into thermal energy and that its dissipation depends on the solvent used and the volume [20], it is necessary to calculate the actual transmitted power (Pa) (Equation (4)). The temperature of the medium was measured manually with a digital thermometer (Gulton do Brasil, Gulterm 180, São Paulo, SP, Brazil) at least every 10 s for 300 s at a distance of 1.5 cm from the ultrasound probe and 2.5 cm in depth in the extraction medium (solvent + SCG).

(4) $$P_{a}=mC_{p}\frac{dT}{dt}$$

where *P_a_* is the actual transmitted power (W), *m* is the total mass of the extraction medium, *T* is the temperature (K), *t* is the time (seconds), and *C_p_* is the specific heat of the medium at constant pressure (J·kg^−1^·K^−1^).

The specific heat of the extraction medium was estimated by a simple mixing rule considering the SCG:solvent ratios of each system and the water content in ET6. The specific heat of the SCG was estimated at 1915.6 J·kg^−1^·K^−1^ [27] considering the composition of the SCG in terms of moisture, proteins, carbohydrates, lipids, and ash. The specific heats of ET0 and water were 2438.46 and 4182.0 J·kg^−1^·K^−1^ [27,28].

In addition, the acoustic energy density (AED) was calculated by dividing *P_a_* by the extraction volume (W·L^−1^), and the transmitted power intensity (PI) was calculated by Equation (5) [20].

(5) $$UI=\frac{4\cdot P_{a}}{\pi D^{2}}$$

where PI is the power intensity (W·cm^−2^) and D is the diameter of the ultrasound probe (cm).

#### 2.3.1. SCGO Extractions in Batch

The UAEs in the batch configuration (designated U4) were performed in an 800-mL Pyrex glass cell jacketed in a thermostatic bath (Marconi, MA184, Piracicaba, SP, Brazil). For these experiments, an SCG:solvent (ET0 or ET6) mass ratio of 1:4, magnetic stirring of 800 rpm, and temperature of 50 ± 9 °C (the temperature was monitored with the aid of a skewer-type thermometer directly in the phase every 5 min of extraction) were used for extraction. The system composed of SCG and solvent was subjected to the application of ultrasound with nominal power intensities of 200 and 600 W (ultrasound probe submerged in 2.1 cm in the extract phase) for 30 min. This time interval was defined based on the study of the SCGO extraction kinetics performed by Toda et al. [14] and preliminary study of UAE kinetics shown in Figure S1 in the Supplementary Material, and this time interval was defined based on the study of the SCGO extraction kinetics performed by Toda et al. [14]. Experiments were also performed considering the same configuration but without ultrasound application. After this period, samples of the extract and raffinate phases were collected for characterization, as described in Section 2.5.

#### 2.3.2. Extractions of SCGO in the Fixed Bed Column with Extract Recirculation

The UAEs in a fixed bed column with recirculation of the extract phase (designated U15) were performed in a continuous flow cell made of stainless steel (high volume continuous flow cell, Sonics and Materials, Newtown, CT, USA), hermetically sealed, jacketed, and connected to a thermostatic bath (Marconi, MA184, Piracicaba, SP, Brazil). Monitoring of the temperature inside the extraction cell was performed with a skewer-type digital thermometer inserted in a T tube connected to silicone hoses at the bottom of the vessel. During the extractions, the extract phase was pumped with the aid of a digital peristaltic pump (Cole Parmer Masterflex L/S, Model 77200-60, Vernon Hills, IL, USA), with a flow rate of 140 mL·min^−1^, through hoses, from the lower region to the upper region of the extraction vessel. The hoses were thermally isolated. A polypropylene filter was placed between the inside of the vessel and the lower lid to prevent solid SCG particles from being transported by the extract phase stream. A known mass of SCG was inserted into the cylindrical vessel together with the solvent (ET0 or ET6) in an SCG:solvent mass ratio of 1:15. Subsequently, the vessel was closed with the ultrasound probe at the top (probe submerged 6 cm in the extract phase). The temperature of the extract phase at the cell outlet was monitored throughout the experiment and was 45 ± 4 °C. The application of ultrasound with nominal power intensities of 200, 400, and 600 W was initiated simultaneously with the activation of the peristaltic pump. After 30 min of extraction, the entire extract phase was removed from the system, the solid phase was collected from the cylindrical vessel, and both phases were immediately subjected to characterization analyses. Experiments were also performed considering the same configuration but without ultrasound application.

### 2.4. Extraction of SCGO Using Pressurized Ethanol

PLEs were performed in a Dionex extractor (ASE 150, Sunnyvale, CA, USA), using ET0 and ET6 as solvents, with a constant pressure of 10.35 MPa, using the procedure detailed by Toda et al. [14]. The SCG:solvent mass ratio was 1:4. The extractions were performed with a contact cycle for 30 min at 50 ± 1 °C. After the pre-established extraction time, the extract and raffinate phases were removed and subjected to characterization.

### 2.5. Characterization of the Products Obtained from the SCGO Extraction Processes

As explained in Section 2.3 and Section 2.4, the extract phases obtained from extractions assisted or not by ultrasound and with pressurized solvent were collected, diluted with hexanol (1:1 mass ratio) to maintain the homogeneity of the phase, and analyzed in terms of solvent by evaporation in an oven with forced convection at 100 °C for 24 h and water content by the Karl Fischer titration method (Ca 2e-84, [21]).

The raffinate phases were analyzed in terms of residual oil content through hexane extraction at 90 °C for 1 h (Ankom, XT 10, Macedon, NY, USA). The characterization of the raffinate and SCG phases enabled the calculation of the extraction yield of SCGO according to Equation (6).

(6) $$\mathrm{SCGO}\;\mathrm{extraction}\;\mathrm{relative}\;\mathrm{yield}\;\left(Y_{\mathrm{SCGO},\;}\%\right)\;=100\times \;(M^{\mathrm{SCG}}\left(w_{\mathrm{SCGO}}^{\mathrm{SCG}}\right)\;-\;M^{\mathrm{DS}}\left(w_{\mathrm{SCGO}}^{\mathrm{DS}}\right))/(M^{\mathrm{SCG}}\left(w_{\mathrm{SCGO}}^{\mathrm{SCG}}\right))$$

where w_SCGO_ is the mass fraction of SCGO in the SCG or defatted solids (DS). M^DS^ and M^SCG^ represent the masses (g) of DS and SCG, respectively.

The raffinate phases were also characterized in terms of the mean particle diameter ($d_{avg})$ based on the particle size distribution from Tyler sieves ($d_{s})$ and laser diffraction ($d_{ld})$, true density ($\rho_{t})$ and apparent density ($\rho_{a}),$ X-ray diffraction, and scanning electron microscopy. All characterizations were performed at least in duplicate, as previously described in Section 2.2.

### 2.6. Designing of the Extractor Configured in Cross-Currents

**Figure 1:**
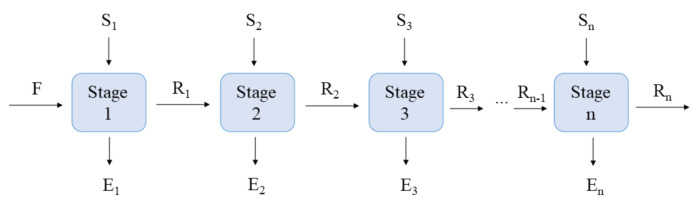
Diagram representing the flow direction of each theoretical stage in an extractor configured in cross-currents.

To better evaluate the performance of the sonication and pressure in the SCGO extraction processes, cross-chain extractors were designed. In the cross-flow configuration, after the first contact of the solvent with the SCG, the partially DS become the feed of the subsequent stage. These partially DS come into contact with new solvent and so on until the desired yield is reached [29]. Figure 1 shows a generic representation of a solid–liquid extraction process configured in cross-currents, in which the feed stream F is the SCG, which contains inert solids (represented by component a, with all compounds being insoluble in the extraction solvent) and soluble solids (represented as component c, SCGO). The currents S_i_ (i is the corresponding stage number) contain only the solvent (component b, ET0 or ET6). The compositions of the output currents of stage i, the raffinate phase (R_i_), and extract phase (E_i_), are related through equilibrium equations. The extract phase is composed of solvent and SCGO (y_bEi_ + y_cEi_), free of inert solids, for any stage i, and the raffinate phase is composed of inert solids and the adhered solution (solvent and SCGO) (x_aRn_ + x_bRn_ + x_cRn_).

Information on the input currents, equilibrium relationship of the SCGO between the extract and raffinate phases, and the solution retention index in the raffinate phase are essential to estimating the number of theoretical stages. In this study, the information on the relative yield of SCGO extraction (Equation (6)), experimentally determined for each type of extraction experiment, was considered the equilibrium ratio of the SCGO between the phases. For all stages in the same extraction system (conventional, UAE, or PLE), the same extraction yield values were considered.

The retention index (R*) is the content of bonded solution (x_bRn_ + x_cRn_) relative to the insoluble solids (kg of bonded solution/kg of inert solids) (Equation (7)).

(7) $$R^{*}=\frac{x_{\mathrm{bRn}}+{\;x}_{\mathrm{cRn}}}{x_{\mathrm{aRn}}}$$

The composition of the raffinate phase is given by Equation (8).

(8) $$x_{\mathrm{aRn}}+{\;x}_{\mathrm{bRn}}+{\;x}_{\mathrm{cRn}}=1$$

The mass fraction of component a in the raffinate phase can be expressed by Equation (9) by the combination of Equations (7) and (8).

(9) $$x_{\mathrm{aRn}}=\frac{1}{1+R^{*}}$$

The retention index values of each extraction system (conventional, UAE, or PLE) were determined experimentally and considered constant between the stages. In the stages subsequent to Stage 1, it was considered that the solvent flow rate (S_2_, S_3_...S_n_) was numerically equal to the flow rate R_i_, thus maintaining a solid:solvent ratio of 1:1 in the contact stages subsequent to Stage 1 (Stages 2, 3, …, n).

Considering this information and establishing a desirable yield value of SCGO extraction at the end of the process or the maximum amount of oil that could be lost in the raffinate phase (x_cRn_), it is possible to calculate the currents E_i_ (y_bEi_, y_cEi_) and R_i_ (x_aRi_, x_bRi_, x_cRi_) by the global mass balance and by the mass balances for the components. In addition, it is possible to estimate the total extract flow (E_total_) and its composition (y_bEtotal_, y_cEtotal_), calculated by combining the extracts from the n extraction stages.

### 2.7. Statistical Analysis

The mean results of the experiments were evaluated by analysis of variance (ANOVA) and Duncan’s multiple range test (DMRT) for comparison of values (*p* ≤ 0.05) using SAS^®^ (Version 9.3, SAS Institute Inc., Cary, NC, USA).

The relationships among the mean particle diameter (d_avg_), mean diameter of the bottom tray particles (d_ld_), true density (ρ_t_), apparent density (ρ_a_), soluble solids content present in the extract phase (SS), and relative extraction yield of SCGO (Y_SCGO_) were investigated by multivariate analysis of the principal components (PCA, principal component analysis) using PAST software, version 4.05 [30]. As the variables were measured in different units, the results were normalized by calculating the difference between the results and the mean values divided by the standard deviation.

## 3. Results

The SCG used in the extractions showed 8.2 ± 0.1% moisture, 23.4 ± 0.2% lipids, 16.2 ± 0.2% crude protein, 0.32 ± 0.03% ash, 0.41 ± 0.04% soluble fiber, and 64 ± 1% insoluble fiber. This chemical composition is in agreement with the values presented by Toda et al. [14]. It is possible to verify that the sum of the components of the SCG is greater than 100%, probably due to the possible variations that the protein conversion factor may present. The variation in the conversion factor can also cause variations in the fiber contents. The methodology used to determine the fiber content considers that most of the proteins are not removed when using this method [23]. Insoluble fibers are the major component of SCG, which has the following composition: 26 ± 1% cellulose, 22 ± 2% hemicellulose, 1.27 ± 0.00% soluble lignin, and 56.35 ± 0.00% insoluble lignin. The sum of the insoluble fiber components is also greater than 100%, probably due to the hydrolysis factors considered to estimate the hemicellulose and cellulose contents, which may vary depending on the material analyzed. Kovalcik et al. [31] reported that SCG contain a high hemicellulose content (30 to 40%) and a lower cellulose content (10%), while Cubas et al. [17] reported a higher cellulose content (45 ± 3%) than hemicellulose (28 ± 7%). These differences in the composition of the SCG may be due to differences in the processes that obtain the SCG as well as the origin of the green coffee beans [31].

Table 1 shows the results of the relative extraction yield of SCGO ($Y_{\mathrm{SCGO}\;},\%)$, soluble solids content in the extract (SS), residual oil content in the raffinate phase (RO), and the retention index (R*) values for the extractions with and without the application of ultrasound, performed in batch (U4) and in a fixed bed column with extract recirculation (U15), and the PLEs.

The retention index values showed no statistically significant differences (*p* > 0.05) in relation to the different solvents used (ET0 and ET6). The extractions with pressurized liquid led to lower R* values, probably due to the depressurization of the system at the end of the extraction process. Regarding the lower R* values for U15, the configuration of the UAE system may have facilitated the drainage of the extract and led to lower retention of solution in the raffinate phase. Similar results were obtained in palm fiber oil extraction comparing batch and fixed bed packed column extractor configurations [32]. Table 1 shows that the application of ultrasound did not cause differences in the retention index values.

According to the results of Table 1, under all conditions, the ET0 solvent showed better performance than ET6, with higher oil extraction yields and soluble solids contents in the extract and, consequently, lower residual oil contents present in the raffinate phase of the extract.

In the UAEs, the values of actual transmitted power (*P_a_*) ranged from 23.07 ± 0.01 to 73 ± 4 W, the power density ranged from 75.86 ± 0.01 to 247 ± 14 W·L^−1^, and the ultrasonic power intensity (UI) ranged from 4.70 to 14.90 W·cm^−2^. Table S1 in the Supplementary Material shows the values and statistical analysis of these determinations.

The fixed bed column extraction experiments (U15) showed higher values of SCGO extraction yield and lower levels of soluble solids in the extract than the batch extractions (U4), regardless of the type of solvent, ET0 or ET6, and the ultrasound power applied (*p* < 0.05). These results are due to the higher ratio of solvent to SCG, 1:15, compared to the solid:solvent ratio of 1:4 used in batch extractions.

Perrier et al. [33] and Sicaire et al. [20] evaluated the UAE of canola oil using hexane, ethanol, and isopropanol as solvents and different liquid/solid ratios. The authors reported that the higher the ratio of liquid to solid and the intensity of ultrasound applied to the system was, the higher the oil extraction yield. Le et al. [34] and Rocha et al. [19] attributed the increase in SCGO extraction yield to the increase in solvent supply and not necessarily to the sonication effect, which is also in agreement with this study. Increasing the solid:solvent ratio from 1:4 to 1:15 increases the driving force of SCGO extraction [35]. Although the performance of ET6 is worse than that of ET0 when comparing the same solid:solvent ratio, comparing the extraction yields of SCGO with ET6 and higher ratio (U15) and ET0 with a lower ratio (U4), the yields are similar. This demonstrates that the use of a greater amount of ET6 in relation to SCG can lead to extraction yields of SCGO compatible with those obtained with ET0.

The experiments performed with pressurized solvent resulted in lower residual SCGO contents in the raffinate phase and, consequently, higher extraction yield values than the experiments performed with the same solid:solvent ratio of 1:4, with or without the application of ultrasound (*p* ≤ 0.05). However, in relation to the soluble solids content in the extract, the extractions with ET0 in the U4 600 W condition and with pressurized ET0 resulted in statistically equal values (*p* > 0.05) that were higher than those of the conventional extraction (U4 0 W). Different results were reported by Toda et al. [14]. These authors, when comparing the results of soluble solids content in the extract for conventional extraction with those of pressurized solvent, did not notice statistically significant differences in extraction temperatures below the normal boiling point of the ET0 and ET6 solvents.

The processes of PLE and UAE are emerging technologies that can intensify the extraction process and accelerate the mass transfer from the solid to the liquid [14,20]. The application of ultrasound waves in liquid medium causes the formation of microbubbles that oscillate and grow until they collapse, resulting in local implosions with high temperature and pressure. These implosions, when they occur on the surface of solids, can cause the rupture of cell walls, improving the diffusion of compounds through the medium and increasing mass transfer [20,36]. In the case of PLE, the extraction can be performed at high temperatures exceeding the boiling point of the solvent, decreasing its viscosity and helping to break the interactions between the solute with the matrix, improving the diffusion of the compounds. High pressure is applied to the extraction cell to maintain the solvent in a liquid state. In the present study, performed at a temperature lower than the normal boiling temperature of ethanol, the pressure can accelerate the penetration of the liquid into the solid matrix, allowing a fast process performance [11,37]. The use of PLE and ultrasound (U4 600 W) for ET0 resulted in soluble solid contents in the extract statistically equal to and higher than those provided by conventional extraction (*p* ≤ 0.05), demonstrating improvement in extraction with the application of these technologies. Unlike the results of this study, Dong et al. [38] obtained a higher extraction yield of green coffee oil with the application of ultrasound than with pressurized liquid.

In a comparison only between the extraction experiments under atmospheric pressure with and without ultrasound and with a solid:solvent mass ratio of 1:4 (U4), the oil extraction yield and the soluble solids content increased with an increasing power applied in the system (*p* ≤ 0.05). Regarding the residual oil content in the raffinate phase, no statistically significant differences were observed due to an increase in ultrasound power (*p* > 0.05).

Unlike the results indicated in the literature [20,33,39], in this study, the effect of ultrasound on the extraction yield of SCGO was not fully evidenced in the set of experiments performed. This motivated the characterization of the solid phases in terms of their morphology in order to better understand which effects lead to increased SCGO extraction yield, solid/solvent ratio, cavitation caused by the action of ultrasound, or pressurization of the solvent.

### 3.1. Morphological Characterization of the Solid Phases from the Extraction Processes

Table 2 shows the results of the mean particle diameter (d_avg_, µm); mean diameter of the bottom tray particles, smaller than 297 µm (d_ld_, µm); true density (ρ_t_, g·cm^−3^); and apparent density (ρ_a_, g·cm^−3^) of the SCG and of the solid phases from the extractions.

When using ET0 as the solvent and U15 0 W and U15 200 W, DS with higher values of d_avg_ were obtained. The same was observed when using ET6 in U15 400 W and U15 600 W UAEs. The lowest d_avg_ values were obtained in the pressurization condition for ET0; however, when using ET6, the lowest values were obtained in the U4 200 W condition. The results were significantly different but did not present a defined standard behavior with respect to the different extraction conditions. In a study conducted by Sumere et al. [16], the particle size of pomegranate peels was an important factor for the UAE of phenolic compounds. The authors did not observe the effect of sonication on the results using raw material with smaller particles (680 µm) compared to larger particles (1050 µm).

**Figure 2:**
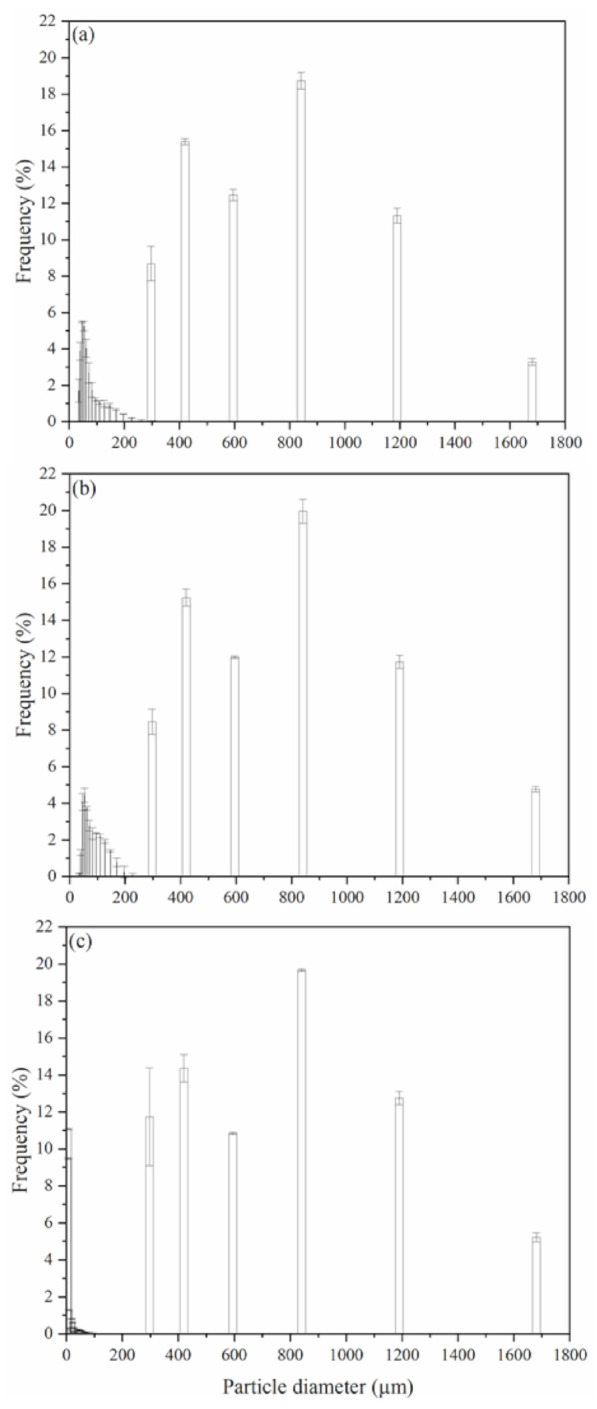
Particle size distribution: (**a**) SCG; (**b**) raffinate phases from extractions without ultrasound (U4 0 W); (**c**) raffinate phases from ultrasound extractions of 600 W power (U4 600 W).

Regarding the diameter of the particles of the bottom tray (d_ld_, µm), Table 2 shows that the raffinate phases from the extractions with ultrasound with the solid:solvent ratio 1:4, regardless of the type of solvent (U4 ET0 200 W, U4 ET0 600 W, U4 ET6 200 W, and U4 ET6 600 W), showed statistically lower values (*p* ≤ 0.05). Based on the observation of the particle size distribution of the SCG particles (Figure 2a) and the raffinate phases of extractions without ultrasound (Figure 2b) and with the application of 600 W of nominal power (Figure 2c), the application of sonication led to an increase in the frequency of smaller diameter particles (close to 8 µm) and the number of particles approximately 300 µm in diameter. These results indicate that there was possibly a breakdown of the oilseed matrix due to the effect of ultrasound. According to Zhao et al. [40], the use of ultrasound leads to the disruption of plant cells, allowing the substances present within the cells to be more easily released. This fact may have occurred with the raffinate phases obtained from the detached extractions (U4 ET0 200 W, U4 ET0 600 W, U4 ET6 200 W, and U4 ET6 600 W). In fact, according to Table 1, the extraction yields of SCGO in condition U4 600 W, for both solvents ET0 and ET6, were statistically higher than those of condition U4 0 W.

Regarding the true density and apparent density, according to Table 2, the raffinate phases had significantly higher values of real density than the SCG. This is probably due to the lower amount of oil in the raffinate phases than in the SCG, considering that the oil has a lower density than the other components of the solid matrix [41]. Comparing the actual density results of the raffinate phases, we can observe that these values are significantly different but do not show standard behavior that can be related to the different extraction conditions used. The same can be observed when comparing the apparent density values, also shown in Table 2.

In general, a comparison of the mean particle diameter values with the apparent density for the raffinate phases from the extractions with ET0 shows that the apparent density increases with decreasing average particle size; the same was reported by Sohn and Moreland [42].

To better understand the effect of ultrasound and pressurization of the system on SCGO oil extraction, Principal Component Analysis (PCA) was performed considering the results of the characterizations shown in Table 2 for mean particle diameter (d_avg_), mean particle diameter of the bottom tray (d_ld_), true density (ρ_t_), and apparent density (ρ_a_), in addition to the extraction yield of SCGO (Y_SCGO_) and soluble solids content present in the extract phase (SS) (Table 1).

**Figure 3:**
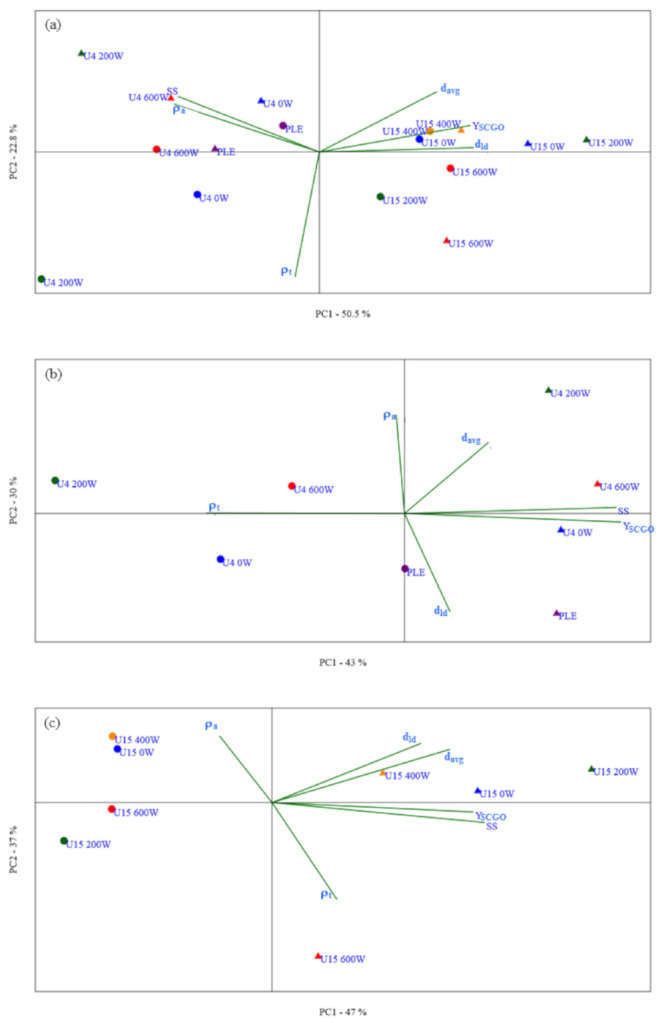
PCA diagram of the SCGO extraction yield (Y_SCGO_), soluble solids content in the extract phase (SS), mean particle diameter (d_avg_), mean diameter of the bottom tray particles (d_ld_), true density (ρ_t_), and apparent density (ρ_a_) of the raffinate phases obtained from extractions: (**a**) assisted or not by ultrasound (0, 200, 400, or 600 W) for batch systems (U4), fixed bed column systems with extract recirculation (U15) and PLE; (**b**) U4 and PLE and (**c**) U15. Solvents: ET0 (▲); ET6 (●).

Considering all the experimental conditions in the PCA, as shown in Figure 3a, the variances of Principal Component 1 (PC1, 50.5%) and Principal Component 2 (PC2, 22.8%) represent 73.3% of the total variance. This result indicates a good correlation between the extraction conditions and the characteristics of the raffinate phases and oil extraction yield.

In Principal Component 1 (PC1), the most representative characteristics of the higher load were the mean diameter of the bottom tray particles (d_ld_) (0.48), extraction yield (Y_SCGO_) (0.47), and mean particle diameter (d_avg_) (0.37). In Principal Component 2 (PC2), the mean particle diameter (d_avg_) (0.38), soluble solids content in the extract (SS) (0.35), and apparent density (ρ_a_) (0.30) were the most representative characteristics (Figure 3a).

Figure 3a shows the diagram of the PCA for the different results of extractions assisted or not by ultrasound or pressurized solvent. In the positive quadrants of the *x*-axis, the representative points of the systems with a solid/solvent mass ratio of 1:15 (U15) were present, which resulted in higher values of Y_SCGO_, d_avg_, and d_ld_, characteristics with higher loads in PC1.

The increase in the solid:solvent ratio from 1:4 to 1:15 resulted in higher extraction yields, as shown in Table 1, due to the greater amount of solvent in the extraction and the consequent increase in the force for mass transfer. Higher extraction yields were obtained for U15, regardless of solvent hydration. In this situation of a high amount of solvent in relation to solid, the use of ultrasound allowed an increase in extraction yield by 200 W for ET0 and 600 W for ET6 compared to those of the conditions without the application of ultrasound (0 W).

The mean particle diameter (d_avg_) and mean diameter of the bottom tray particles (d_ld_) of the raffinate phases from the U15 extractions were similar to the particle sizes of the raw material (SCG) and larger than the particles from the U4 extractions. Based on these observations, it can be inferred that in extractions with a greater amount of solvent, a solid solid:solvent ratio of 1:15, the increase in oil extraction yield was mainly due to the increase in driving force and not due to ultrasound cavitation, since the diameters d_avg_ and d_ld_ were minimally modified.

In general, the use of pressurized solvents ET0 and ET6 led to higher extraction yields than the U4 extractions with and without the use of ultrasound.

A greater influence of the process conditions on the apparent density of the raffinate phases from the UAEs can be observed for systems with solid:solvent ratios of 1:4. In general, the extractions with ultrasound (U4 200 W and U4 600 W) resulted in higher apparent density values, which may indicate the presence of a larger number of smaller diameter particles. These observations may indicate that the application of ultrasound led to the breaking of the particles in the extraction process at 1:4 solid:solvent ratios. However, this break did not allow an increase in oil extraction yield, regardless of the solvent used and the ultrasound power applied.

Goh et al. [4] obtained better results of SCGO extraction at 150 W ultrasound amplitudes using hexane as the extraction solvent and a liquid:SCG ratio of 4 mL·g^−1^ for 30 min. The authors reached a maximum extraction of 14.55% (yield calculated based on the content of soluble solids extracted in relation to the initial mass of SCG). The content of soluble solids extracted was higher than the SCGO content obtained using the Soxhlet extraction method with the same solvent (12.5%), which may indicate the extraction of nonlipid components. In a study by Gerde et al. [15], the authors observed the extraction of intracellular material with the oil when higher sonication energy was used in the processing of microalgae. Depending on the conditions and species of microalgae, even after cell wall rupture, some membranes tend to retain lipids [43]. In fact, in the U4 200 W and U4 600 W experiments, there was an increase in soluble solids in the extract, but there was no concomitant increase in the relative yield of SCGO. These results demonstrated that cavitation may have released other nonlipid components of the coffee ground matrix.

PCA was also performed for the different sets of extraction experiments for the different ratios of solid:solvent evaluated, U4 and U15. These analyses are shown in the diagrams of Figure 3b,c, respectively.

For U4, Principal Component 1 (43%) showed higher loads in relation to the extraction yield (Y_SCGO_) (0.57) and soluble solids content in the extract (SS) (0.57). In PC2 (30%), the apparent density (ρ_a_) (0.63) and mean particle diameter (d_avg_) (0.45) were the characteristics with the highest representation. Figure 3b shows that in the positive quadrant of the *x*-axis, all points were representative of the extractions performed with ET0 as the solvent, which resulted in higher extraction yields of SCGO (Y_SCGO_) and soluble solids content in the extract phase (SS). In the positive quadrants of the *y*-axis, all the representative points of the UAEs (U4 200 W and U4 600 W) with higher apparent density values (ρ_a_) and mean particle diameter (d_avg_) were present, corroborating a possible effect of ultrasound application of increasing the frequency of smaller particles (Figure 2).

In the case of U15, PC1 (47%) had the soluble solids content in the extract (SS) (0.55), extraction yield (Y_SCGO_) (0.52), mean particle diameter (d_avg_) (0.46), and mean diameter of the bottom tray particles (d_ld_) (0.39) as the main characteristics. In PC2 (37%), the apparent density (ρ_a_) (0.56), mean particle diameter of the bottom tray (d_ld_) (0.38), and mean particle diameter (d_avg_) (0.34) were the most representative characteristics (Figure 3c). In Figure 3c, it is possible to observe the same behavior of the extraction solvent with U4 (representative points of extractions with ET0 in the positive quadrant of the *x*-axis) with higher Y_SCGO_, SS, d_avg_, and d_ld_. However, unlike U4 extractions, U15 extractions did not seem to show differences in behavior regarding the application of ultrasound waves.

**Figure 4:**
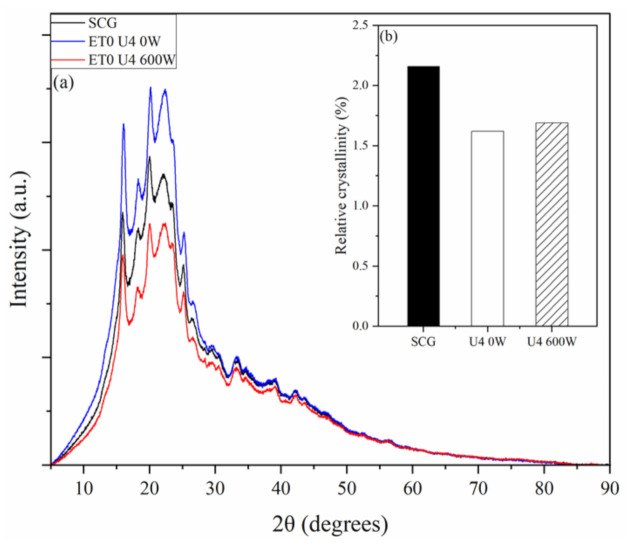
(**a**) X-ray diffractograms and (**b**) relative crystallinity (%) of SCG and the solid phases obtained from batch extractions (U4 0 W and U4 600 W).

The SCG and the solid phases obtained from batch extractions (U4 0 W and U4 600 W) were analyzed by X-ray diffraction (XRD), according to the diffractograms shown in Figure 4. In general, the three materials showed diffraction patterns similar to each other and similar to the SCG diffractograms presented by Ballesteros et al. [5] and Chien et al. [44].

The deconvolution of the peaks (determination coefficients greater than 0.9994) was performed in the diffractograms of Figure 4. Ballesteros et al. [5] used the cellulose standard of the International Center for Diffraction Data (ICDD 00-003-0226) as a reference and found that the cellulose standard was similar to the XRD standard of the SCG. Chien et al. [45] showed that the crystalline peak in the SCG at 2θ of 22° corresponded to the XRD of cellulose. Gabriel et al. [45] reported that for all forms of cellulose I evaluated in the study, including the XRD of coffee husks, the peak clarity at approximately 22° indicated increased crystallinity.

Perdana et al. [46] showed that the peaks found in the XRD of roasted coffee beans correspond to the peaks of the XRD standard of crystalline sucrose (2θ = 11.443°, 18.842°, and 24.773°) (ICDD 00-024-1977). According to Wei et al. [47], sucrose is one of the main sugars present in green coffee and is degraded into glucose and fructose during the thermal treatment of bean roasts. Considering that, to obtain the SCG, the green coffee beans were subjected to different thermal and extraction processes, the SCG probably did not contain sucrose in their composition. Toda et al. [14] determined the composition of SCG in terms of sugars, and they were composed of mannose, fructose, galactose, glucose, inositol, and arabinose.

Based on this information, to verify possible changes in the SCG crystallinity by ultrasound application, the deconvolution peak at approximately 2θ of 22° was considered the crystalline peak. Thus, it was possible to estimate the relative crystallinity, as shown in Figure 4b. Considering these values, it can be inferred that the application of ultrasound did not cause changes in the crystalline structure of the SCG.

**Figure 5:**
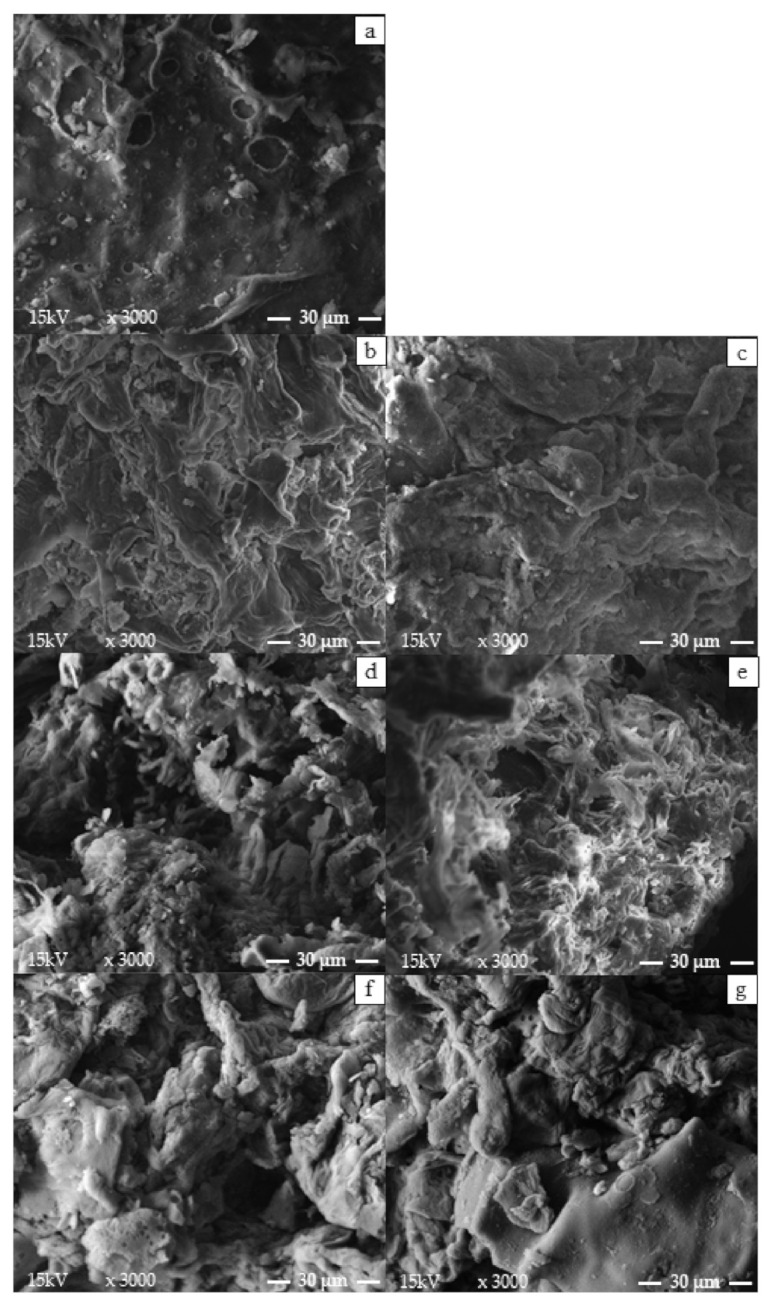
Scanning electron microscopy (SEM) images (magnified 3000 times in high vacuum) of (**a**) SCG andthe raffinate phases from the extractions using (**b**) ET0 without ultrasound U4 0 W, (**d**) extractions with ultrasound (U4 600 W), and (**f**) PLE. Scanning electron microscopy (SEM) images (magnified 3000 times in high vacuum) of the raffinate phases from the extractions using (**c**) ET6 U4 0 W, (**e**) U4 600 W, and (**g**) PLE.

Scanning electron microscopy (SEM) images (magnified 3000 times in high vacuum) are shown in Figure 5 of the SCG and the raffinate phases from the extractions without ultrasound (U4 0 W), with ultrasound (U4 600 W), and using ethanol PLE with ET0 or ET6.

Although XRD does not allow the confirmation of the possible effects of the application of ultrasound, in Figure 5, it can be observed that the use of ultrasound caused changes in the surface of the solid, most likely due to the effect of cavitation. These observations corroborate the results of the lower particle diameter values of the bottom tray (d_ld_), which were associated with the breakdown of the oilseed matrix by the ultrasound effect. In a study conducted by Okur et al. [18], it was possible to visualize significant differences when comparing the SEM images of conventional extraction and UAE. The authors used a solution with 80% methanol to recover phenolic compounds from SCG. Differences in micrographs were also observed in studies presented by Cubas et al. [17] for ultrasound-assisted SCGO extraction and Ravindran et al. [48], who used the application of ultrasound as a pretreatment of SCG for cellulose and hemicellulose recovery.

In the case of PLE, it is possible to visualize openings in the solid surface, which may be due to the path of the pressurized solvent in the SCG. Cellular damage was also observed with the high pressure and temperature of the PLE for oil extraction from ground green coffee beans [38], with micrographs similar to those shown in Figure 5. For the raffinates obtained with solid:solvent ratios of 1:15, no striking differences were observed in the structures of the materials in relation to the SCG, reinforcing that the higher extraction yields were due to the greater amount of solvent in relation to the SCG.

Regarding the micrographs, both UAE and PLE caused changes in the surface of the raffinate phases. However, the application of ultrasound did not cause changes in the crystalline structure of the SCG, as observed through XRD analysis.

In general, sonication of the medium led to higher values of apparent density of the raffinate phases, with the presence of a larger number of particles of smaller diameter. In addition, these effects of the application of ultrasound were observed only in systems with a higher ratio of solid to solvent (U4). In the case of pressurization of the system, these effects on the apparent density values and particle size were not observed.

### 3.2. Designing of the Extractor Configured in Cross-Currents

**Figure 6:**
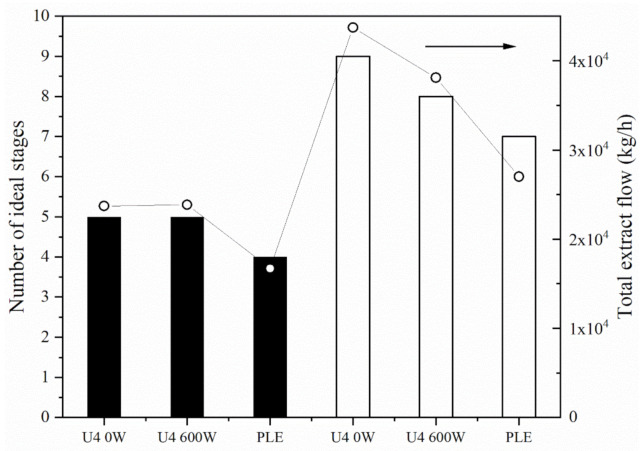
Number of ideal stages in a continuous extractor configured in cross currents and total extract flow as a function of extraction conditions. Bars: Number of ideal stages; (○) total extract flow (Etotal). Simulations using ET0 (filled bars) and ET6 (empty bars).

The number of ideal stages of continuous extractors configured in cross-currents necessary to achieve 99% SCGO extraction yield was calculated for each extraction system, conventional (U4 0 W), UAE (U4 600 W), and PLE. The total extract flow rate (E_total_), the sum of all extracts obtained, and the total extract composition in terms of SCGO were also estimated. The results of these calculations are shown in Figure 6 for solvents ET0 and ET6. For the calculations, it was considered that the feed flow rate (F) of SCG was 2000.0 kg·h^−1^ and that the SCG oil composition was 23.4 g·100 g^−1^ dry SCG (x_cF_ = 0.2163 on a wet basis), and the retention indices were determined experimentally and are shown in Table 1.

Figure 6 shows that the best PLE performance was independent of the solvent used, with a lower number of ideal stages than conventional and UAEs. This difference is most likely due to the lower retention index values of the systems with pressurized liquid. Regarding the application of ultrasound, the effect of sonication was observed only for the ET6 solvent. In the results for the number of stages calculated, the total extract flow rate followed the same trend as the number of estimated ideal stages. Sumere et al. [16] obtained better results with sonication, requiring one fewer stage in the UAE. Sicaire et al. [20] also verified the higher efficiency of the application of ultrasound in the extraction of canola oil, observing a decrease in the number of stages in an extractor configured in cross-currents.

**Figure 7:**
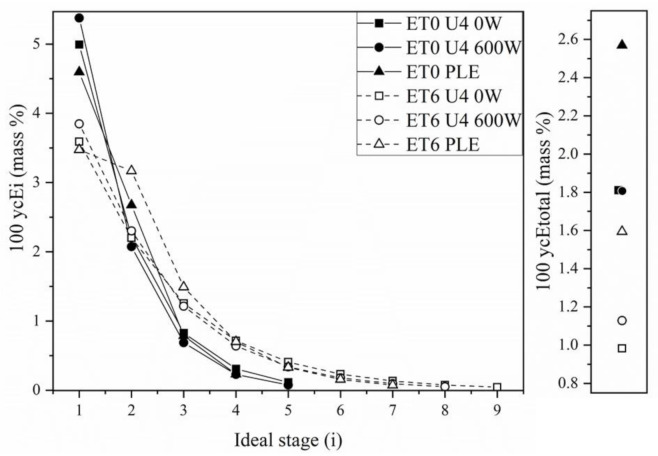
Mass percentages of SCGO in the extracts of each stage (100 ycE_i_, mass %) as a function of the respective ideal stage (i) and mass percentage of SCGO in the total extract (100 ycE_total_, mass %).

The values of mass percentage of SCGO in the extracts of each stage as a function of the respective ideal stage and the mass percentage of SCGO in the total extract can be seen in Figure 7. One of the most important factors for obtaining economically viable extraction processes is the higher concentration of SCGO in the final extract. Thus, we verified that the systems that used ET0 as the extraction solvent resulted in final extracts (E_total_) that were more concentrated than the extracts obtained with ET6. Following the same trend as the total extract flow rate, PLE resulted in more concentrated extracts, regardless of the solvent used.

A comparison of the mass percentages of SCGO in the extracts from the first stages, as shown in Figure 7, with the experimental results of the soluble solids content present in the extract phase (Table 1) shows that these data do not follow the same trend. According to Figure 7, the estimated fractions for the first stage of the PLE are smaller than those for the other systems, while the SCGO fractions of the extracts obtained from the UAE are the largest. According to the global and component mass balance ratios, lower retention index values (R*) result in a lower raffinate phase flow rate (R_1_), higher extract flow rate (E_1_), and, consequently, more diluted extracts. Comparing only the results with and without ultrasound, the results shown in Table 1 are in agreement with the results of Figure 7. With respect to the experimental results of the PLE, other compounds were probably extracted concomitantly with the SCGO according to the comparisons between the calculated values and experimental values.

In general, extraction of SCGO with pressurized ethanol proved to be slightly more advantageous than that with UAE. However, the low concentration of SCGO in the final extracts, for all conditions studied, does not favor the use of this type of extractor configured in cross-currents.

## 4. Conclusions

Ultrasound-assisted SCGO extraction was performed with two different SCG:solvent ratios (U4 and U15) and solvent hydration levels (ET0 and ET6) and various ultrasound powers (200, 400, and 600 W). Extractions performed only with agitation and with pressurized solvent were also performed for comparison purposes.

Under all conditions, ET0 showed better performance than ET6, with higher values for SCGO extraction yield and soluble solids content in the extract phase. ET6 produced extraction yields similar to those obtained with ET0 (U4) only when greater amounts of solvent in relation to SCG were used (U15).

The fixed bed column extraction experiments (U15) resulted in higher values of SCGO extraction yield and lower levels of soluble solids in the extract than batch extractions (U4), regardless of the type of solvent, ET0 or ET6, or ultrasound power. On the other hand, sonication produced an increase in the extraction yield of SCGO and an increase in the content of soluble solids under a solid:solvent ratio of 1:4 (U4). The morphological characterization of the solid phases obtained from these extractions allowed us to verify that the application of ultrasound in the U4 systems led to smaller diameter particles, which may have been the result of the breakdown of the oilseed matrix by the ultrasound effect.

PCA indicated that the effects of the ratio between the solvent and SCG and solvent hydration overlap the effects of sonication and pressurization at the temperature condition studied (50 °C). In the U4 systems, the application of ultrasound resulted in DS with higher apparent density values, corroborating the greater number of particles of smaller diameter.

The X-ray diffraction analysis, based on the cellulose diffraction pattern, indicated that the ultrasound application did not cause changes in the crystalline structure of the DS. On the other hand, a comparison between the SCG micrographs and the U4 raffinate phases made it possible to visualize changes in the surface of the solids resulting from sonication and pressurization.

In the dimensioning of continuous extractors configured in cross-currents, sonication led to a smaller extractor with the use of ET6; however, for ET0, sonication did not favor a smaller number of ideal stages. In the case of pressurization of the system, it was possible to obtain smaller extractors, allowing greater savings with the amount of solvent and the required extraction time. However, the final extracts were much more diluted than those required industrially.

In general, the results indicate that the intensification techniques by ultrasound and pressurization for the system under study (SCG/ethanol) do not allow significant gains in terms of the yield of SCGO extraction compared to conventional extraction. Aiming to make this extraction process industrially applicable, the evaluation of other extraction configurations can be suggested, such as the use of countercurrent extractors, besides the association of intensification techniques. Further, the study of pilot-scale extraction, the economic feasibility analysis of alcoholic extraction, and deep characterization of SCGO considering minor compounds and aroma profiles are also highlighted as future trends.

## Figures and Tables

**Table 1:** Relative extraction yield of SCGO (Y_SCGO_, %), soluble solids content in the extract (SS), residual oil content in the raffinate phase (RO), and the retention index (R*) values for the extractions with and without the application of ultrasound (0, 200, 400, or 600 W) performed in batch (U4) and in a fixed bed column with extract recirculation (U15), and the PLEs.

	SCGO Extraction Relative Yields (Y_SCGO_, %)		Soluble Solids Content in the Extracted Phase (SS, % Mass)		Residual SCGO Content in the Raffinate Phase (% Mass)		Liquid Holdup (kg of Adhered Solution/kg of Inert Solid)	
	ET0	ET6	ET0	ET6	ET0	ET6	ET0	ET6
U4 0 W	62.7 ± 0.7 ^eA^	43 ± 1 ^eB^	4.07 ± 0.06 ^bE^	2.58 ± 0.02 ^cF^	9.8 ± 0.3 ^aD^	15.3 ± 0.2 ^aC^	1.9 ± 0.1 ^aF^	2.07 ± 0.03 ^aF^
U4 200 W	61.9 ± 0.5 ^eA^	43.5 ± 0.5 ^eB^	4.10 ± 0.05 ^bE^	2.56 ± 0.03 ^cF^	9.9 ± 0.2 ^aD^	15.3 ± 0.2 ^aC^	1.98 ± 0.04 ^aF^	2.1 ± 0.1 ^aF^
U4 600 W	66.9 ± 0.3 ^dA^	47.3 ± 0.2 ^dB^	4.6 ± 0.1 ^aE^	2.67 ± 0.03 ^bF^	9.5 ± 0.3 ^aD^	14.4 ± 0.5 ^aC^	1.95 ± 0.02 ^aF^	1.9884 ± 0.0002 ^aF^
U15 0 W	82.4 ± 0.4 ^abA^	74.99 ± 0.04 ^aB^	1.23 ± 0.02 ^cE^	1.09 ± 0.03 ^dE^	5.3 ± 0.1 ^cdD^	7.4 ± 0.1 ^dC^	1.3 ± 0.1 ^bE^	1.3 ± 0.1 ^bE^
U15 200 W	83 ± 2 ^abA^	74 ± 2 ^aB^	1.24 ± 0.04 ^cE^	1.08 ± 0.03 ^dE^	5.1 ± 0.5 ^dD^	7.8 ± 0.4 ^dC^	1.19 ± 0.03 ^bE^	1.33 ± 0.01 ^bE^
U15 400 W	84 ± 2 ^aA^	74.3 ± 0.8 ^aB^	1.243 ± 0.006 ^cE^	1.052 ± 0.003 ^dE^	4.7 ± 0.5 ^dD^	8.1 ± 0.8 ^cdC^	1.1 ± 0.1 ^bE^	1.24 ± 0.02 ^bcE^
U15 600 W	81.1 ± 0.3 ^bA^	69 ± 1 ^bB^	1.20 ± 0.03 ^cE^	1.07 ± 0.05 ^dE^	5.9 ± 0.2 ^cD^	8.9 ± 0.3 ^cC^	1.13 ± 0.04 ^bE^	1.2 ± 0.1 ^cE^
PLE	70.6 ± 0.1 ^cA^	53 ± 1 ^cB^	4.5 ± 0.1 ^aE^	2.92 ± 0.04 ^aF^	8.2 ± 0.2 ^bD^	12.7 ± 0.2 ^bC^	1.14 ± 0.04 ^bG^	1.170 ± 0.003 ^cG^

Means followed by equal lowercase letters in the same column and equal uppercase letters in the same row, for each evaluated answer, do not differ from each other at the 5% level of significance by the Duncan Test.

**Table 2:** Mean particle diameter (d_avg_, µm), mean diameter of the bottom tray particles, smaller than 297 µm (d_ld_, µm), true density (ρ_t_, g·cm^−3^), and apparent density (ρ_a_, g·cm^−3^) of the SCG and of the solid phases from the extractions with and without the application of ultrasound (0, 200, 400, and 600 W) performed in batch (U4) and in a fixed bed column with extract recirculation (U15), and the PLEs.

	Average Particle Diameter (d_avg_, µm)		Average Diameter of Bottom Tray Particles (d_ld_, µm)		True Density (ρ_t_, g·cm^−3^)		Apparent Density (ρ_a_, g·cm^−3^)	
	ET0	ET6	ET0	ET6	ET0	ET6	ET0	ET6
U4 0 W	798 ± 8 ^bcA^	749 ± 5 ^bcB^	67 ± 1 ^dC^	63 ± 1 ^fC^	1.72 ± 0.01 ^dD^	1.94 ± 0.02 ^bD^	0.442 ± 0.002 ^bD^	0.441 ± 0.003 ^cdD^
U4 200 W	802 ± 1 ^bcA^	729 ± 28 ^cB^	9.4 ± 0.1 ^gC^	8.5 ± 0.1 ^hC^	1.65 ± 0.01 ^eC^	2.41 ± 0.02 ^aC^	0.47 ± 0.01 ^aC^	0.456 ± 0.001 ^bC^
U4 600 W	786 ± 30 ^bcA^	778 ± 8 ^abcA^	8.45 ± 0.05 ^gB^	8.6 ± 0.3 ^hB^	1.71 ± 0.01 ^dB^	1.79 ± 0.01 ^eB^	0.444 ± 0.005 ^bB^	0.443 ± 0.001 ^cB^
U15 0 W	857 ± 14 ^aA^	784 ± 15 ^abB^	92 ± 2 ^bC^	82.6 ± 0.2 ^bC^	1.87 ± 0.02 ^bD^	1.717 ± 0.005 ^gD^	0.430 ± 0.003 ^cdD^	0.44 ± 0.01 ^cdD^
U15 200 W	873 ± 19 ^aA^	750 ± 5 ^bcB^	117.93 ± 0.03 ^aC^	68 ± 1 ^eD^	1.87 ± 0.02 ^bE^	1.90 ± 0.02 ^cE^	0.427 ± 0.004 ^deE^	0.4327 ± 0.0002 ^deE^
U15 400 W	819 ± 1 ^bA^	812 ± 1 ^aA^	76 ± 1 ^cD^	80.3 ± 0.3 ^cC^	1.78 ± 0.02 ^cE^	1.74 ± 0.01 ^fE^	0.438 ± 0.005 ^bcE^	0.439 ± 0.001 ^cdE^
U15 600 W	800 ± 1 ^bcA^	808 ± 48 ^aA^	61.4 ± 0.1 ^eB^	78.4 ± 0.2 ^dB^	2.10 ± 0.01 ^aC^	1.83 ± 0.01 ^dC^	0.42 ± 0.01 ^eC^	0.426 ± 0.001 ^eC^
PLE	696 ± 9 ^dB^	776 ± 8 ^abcA^	76 ± 3 ^cD^	87 ± 2 ^aC^	1.79 ± 0.01 ^cE^	1.698 ± 0.005 ^hE^	0.439 ± 0.004 ^bcE^	0.440 ± 0.004 ^cdE^
SCG	776 ± 12 ^c^	776 ± 12 ^abc^	58.3 ± 0.6 ^f^	58.3 ± 0.6 ^g^	1.52 ± 0.01 ^f^	1.52 ± 0.01 ^i^	0.47 ± 0.01 ^a^	0.47 ± 0.01 ^a^

Means followed by equal lowercase letters in the same column and equal uppercase letters in the same row, for each evaluated answer, do not differ from each other at the 5% level of significance by the Duncan Test.

## Data Availability

The data presented in this study are available on request from the corresponding author.

## Supplementary Materials

The following is available online at https://www.mdpi.com/article/10.3390/foods11040584/s1, Figure S1: Extraction kinetics of total soluble solids from spent coffee grounds (mass% in the extracted phase) using absolute ethanol, solid:solvent mass ratio of 1:5, at 50 °C. Nominal power intensities of (■) 188 W and (□) 611 W. Table S1: Actual ultrasound power (P_a_, W), power density (AED, W·L^−1^), and ultrasonic power intensity (UI, W·cm^−2^) of SCGO ultrasound assisted extractions.
