# Supplementary material for: Evaluation of Techniques for Intensifying the Process of the Alcoholic Extraction of Coffee Ground Oil Using Ultrasound and a Pressurized Solvent
Source: Foods. 2022 Feb 17;11(4):584. doi: 10.3390/foods11040584 (PMC8871055; doi:10.3390/foods11040584)
Supplement: Supplementary file 1 [file foods-11-00584-s001.zip › foods-1547050-supplementary.pdf]

foods

MDPI

Evaluation of Intensifying Techniques for the Alcoholic Extraction Process of Spent Coffee Grounds Oil Using Ultrasound and Pressurized Solvent

Tatiane Akemi Toda [1], Ana Julia Morelli Santana [1], Julieta Adriana Ferreira [2], Eliria Maria de Jesus Agnolon Pallone [2], Claudio Lima de Aguiar [3] and Christianne Elisabete da Costa Rodrigues [1,*]

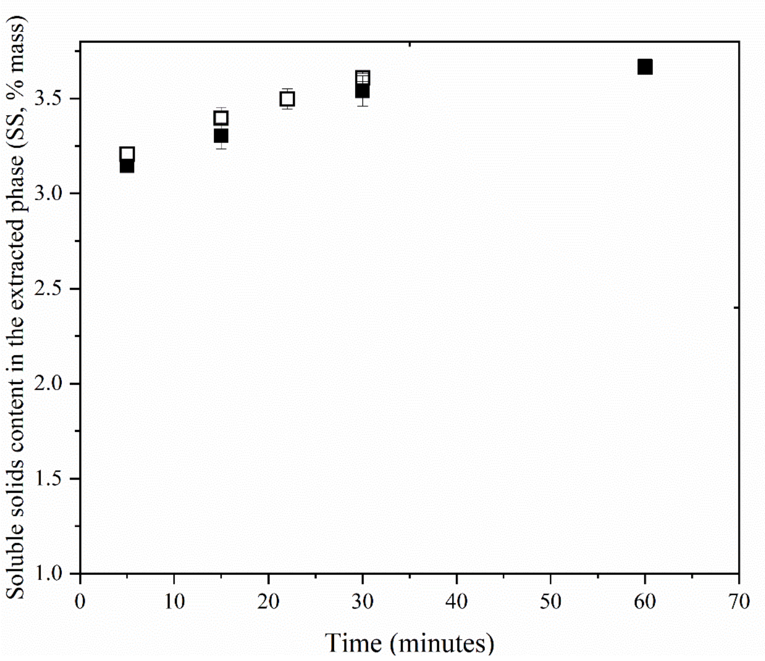

Figure S1. Extraction kinetics of total soluble solids from spent coffee grounds (mass% in the extracted phase) using absolute ethanol, solid:solvent mass ratio of 1:5, at 50 °C. Nominal power intensities of (■) 188 W and (□) 611 W.

Table S1. Actual ultrasound power (P_a, W), power density (AED, W·L^{-1}), and ultrasonic power intensity (UI, W·cm^{-2}) of SCGO ultrasound assisted extractions.

Solvent	Solid:solvent mass ratio	Nominal power (W)	Actual ultrasound power (P_a) (W)[a]	CV[b]	Power density (AED) (W·L^{-1})	CV[b]	Ultrasonic power intensity (UI) (W·cm^{-2}) [c]
ET0	4	200	25 ± 2 c	8.51	84 ± 7 c	8.31	5.06
	4	600	73 ± 4 a	5.89	247 ± 14 a	5.64	14.90
	15	200	23.07 ± 0.01 c	0.06	75.86 ± 0.00 c	0.00	4.70
	15	400	46.15 ± 0.01 b	0.03	151.72 ± 0.00 b	0.00	9.41
	15	600	69.26 ± 0.01 a	0.02	227.57 ± 0.01 a	0.00	14.12
ET6	4	200	24.22 ± 0.01 c	0.04	83.76 ± 0.00 c	0.00	4.94
	4	600	71 ± 2 a	2.97	246 ± 7 a	3.01	14.51
	15	200	23.99 ± 0.01 c	0.04	80.78 ± 0.00 c	0.00	4.89
	15	400	48.03 ± 0.02 b	0.05	161.57 ± 0.00 b	0.00	9.79
	15	600	69 ± 4 a	6.02	232 ± 14 a	6.15	14.05

[a] Calculated by Equation 4; [b] Coefficient of variation; [c] Calculated by Equation 5. Mean values followed by the same lowercase letters in the same column indicate no significant difference by the Duncan's test ($p \leq 0.05$).
